# Sero-prevalence and trends of transfusion-transmissible infections among blood donors at Bahir Dar district blood bank, northwest Ethiopia: A four year retrospective study

**DOI:** 10.1371/journal.pone.0214755

**Published:** 2019-04-11

**Authors:** Elias Shiferaw, Wasihun Tadilo, Iyasu Melkie, Mikru Shiferaw

**Affiliations:** 1 Department of Hematology and Immunohematology, School of Biomedical and Laboratory Sciences, College of Medicine and Health Sciences, University of Gondar, Gondar, Ethiopia; 2 Bahir Dar District Blood Bank, Bahir Dar, Ethiopia; Centre de Recherche en Cancerologie de Lyon, FRANCE

## Abstract

**Background:**

Blood transfusion is an effective treatment for saving millions of lives even though transfusion- transmissible infections are the major problem. Therefore, the aim of this study was to assess the sero-prevalence and trend of transfusion-transmissible infections among blood donors.

**Methods:**

A retrospective study was conducted form July 2014 to June 2018 at Bahir Dar district blood bank. Descriptive statistics was presented using percentages, medians and interquartile ranges. Logistic regression was used to explore risk factors associated with each transfusion transmissible infections.

**Result:**

From a total of 35,435 blood donors 2130 (6.0%) of them had serological evidence for at least one infection and 50 (0.14%) of them were confirmed as having multiple infections. The overall sero-prevalence of HBV, HCV, HIV and syphilis was 230 (6.0%) with 3.9%, 0.6%, 0.5% and 1.2% respectively. From those who had co-infection majority of them 22 (44.0%) were attributed to HBV-Syphilis co-infection and 1 (2.0%) study participant was co-infected with HBV-HIV- Syphilis infection. There was an increment in the overall prevalence of transfusion-transmissible infection;183 in 2014/2015 to 624 in 2017/2018. The sero-prevalence of HBV show a significant increment tend with respect to year of donation. On the other hand HCV and HIV sero-prevalence show an increasing trend from 2014 and decrease in 2018. The sero-prevalence of syphilis was 67 (1.3%) in 2015 and duplicate in 2016, 138 (1.5) but subsequently decrease to 110 (1.1%) in 2017 and in 2018 it was 114 (1.0%).

**Conclusion and recommendations:**

His finding showed growing evidence in the burden of transfusion-transmissible infection in blood donors despite which requires advanced and vigilance screening of donated blood prior to transfusion. More over there should be strategies for monitoring the implementation of post donation counseling for recruitment and retention of safe regular donors.

## Introduction

Blood transfusion is an effective treatment for saving millions of lives worldwide each year. It is a crucial element for the health care service even though, the service is not without risks [1]. Transfusion transmissible infections (TTI) are the major problem which leads to the transmissions of infectious agents from donor to recipient. Common infectious agents include Hepatitis B Virus (HBV), Hepatitis C Virus (HCV), Human Immunodeficiency Virus (HIV) and Syphilis [2].

According to World Health Organization (WHO) 2015 report globally an estimated 257, 71 and 36.7 million peoples were living with chronic HBV, HCV and HIV infections, respectively. The epidemic caused by HBV affects mostly the WHO African Region with prevalence of 6.1% and the Western Pacific Region 6.2% of prevalence. On the other hand HCV infection affects all regions, with a highest prevalence in Eastern Mediterranean Region 2.3% and the European Region1.5% of prevalence. Moreover it was estimated about 1.34 million death were attributed to hepatitis [3].

Transfusion of infected blood is the cause of 5–10% of HIV infections in sub-Saharan Africa. About 12.5% of patients who receive blood transfusions are at risk of post-transfusion hepatitis [4]. In Ethiopia different studies showed the prevalence of TTI. A study from Gondar showed that the total sero-prevalence of TTI among blood donors as 9.5% [5] and 6.55% [6]. On the other hand Jijiga report showed that the sero-prevalence of these infection was 11.5 [7]. Moreover the study from Desie showed that the prevalence of HIV in blood donors was 5.1% [8].

Transfusion of unsafe blood is very costly from both human and economic points of view. It results in morbidity and mortality which have far-reaching consequences, not only for the recipients, but also for their families, their communities and the wider society. Since a person can transmit an infection during its asymptomatic phase, thus transfusions can contribute to an ever-widening pool of infection in the population. The economic costs of the failure to control the transmission of infection include increased requirement for medical care, higher levels of dependency and the loss of productive labor force, placing heavy burdens on already overstretched health and social services and on the national economy [9].

The high prevalence of TTI has heightened the problems of blood safety in Ethiopia. Thus, continuous monitoring of the magnitude and trend of TTI in blood donors is important for assessment of the effectiveness of screening programs. It might also be related directly to the prevalence of the disease in the community. In additions, it provides baseline information for optimizing donor recruitment strategies and post donation counseling service to minimize the transmission of infectious diseases. Therefore, the current study aimed to assess the sero-prevalence and trend of HBV, HCV, HIV and Syphilis among blood donors at the Bahir Dar District Blood Bank, northwest, Ethiopia.

## Material and methods

### Study design and setting

A retrospective cohort study was conducted at Bahir Dar District Blood Bank among blood donors who donated blood during July 2014 (2014/2015) to June 2018 (2017/2018). The Blood Bank is located in the capital city of Amhara Regional state, Bahir Dar city which is 564 km far from Addis Abeba, the capital city of Ethiopia. On average, the blood bank collects 10,000 units of blood annually which provide majority of this blood to Felege hiwot comprehensive referral hospital and up to 17 nearby hospitals.

### Study population

The study population were all blood donors who donated blood at Bahir Dar District Blood Bank with in the study period with screening test for HBsAg, anti-HCV, anti-HIV and syphilis. A total of 35435 blood donors’ record have been reviewed and included in the current study.

### Serological investigations

Serum or plasma samples were tested for HBV, HIV, HCV and syphilis using the Enzyme Linked Immunosorbent Assay (ELISA) (HIV1/2: Vironostika HIV Uni-Form II Ag/Ab fourth generation ELISA, Bio-Merieux, Boxtel, Netherlands; HBsAg: a third generation ELISA, Hepanostika HBsAg UNi-Form II, Bio-Merieux, Boxtel, Netherlands; HCV: Human anti-HCV third generation ELISA, Human Gasellschaft for Bio-chemical and diagnostic MbH, Germany). All tests were performed according to the manufacturer’s instructions.

### Data collection and statistical analysis

Data on socio-demographic variables, laboratory test results were collected from registration book of Bahir Dar District Blood Bank using data extraction format. After checked for completeness the data was entered into Epi Info software (version 7), and then transferred to SPSS version 20 software for analysis. Descriptive statistics were performed, and the results were presented in tables. The sero-prevalence of HBV, HCV, HIV and syphilis were expressed in percentages for the entire study group and groups with different demographic characteristics and donation frequencies. The prevalence of TTIs was determined by the number of donations with positive TTI serologic markers in a year divided by the total number of blood donations in that year. Chi-square trend test was applied to examine the variation in trends. Logistic regression was employed to explore the association between dependent and independent variables. Statistically, P values of below 0.05 were considered as statistically significant.

### Ethical considerations

The study was conducted after ethical clearance was obtained from the School of Biomedical and Laboratory Sciences, College of Medicine and Health Sciences, the University of Gondar.

Permission letter was also obtained from Bahir Dar District Blood Bank. Data were used without using any personal identifier and kept in a confidential manner. Since the study was conducted on a secondary data, an informed consent was not sought from study participants.

## Result

### Socio-demographic characteristics of study participants

A total of 35,435blood donors were screened for transfusion transmissible infections with in a period of four consecutive year at Bahir Dar district blood bank. Majority 23032 (65%) and 26990 (76.2%) of the study participants were males and within 18–24 years, respectively. The median age of the participants was 20 years with a rangeof18–65 years. Mobile donation centers were the place where majority (92.2%) of blood collected. On the other hand almost all 35248 (99.5%) of study participants were volunteer type of blood donors (Table 1).

**Table 1:** Sociodemographic characteristics of blood donors at Bahir Dar district blood bank, northwest, Ethiopia July 2014 to June 2018.

Variables	Category	Frequency	Percentage
**Age**	18–24 years	26990	76.2
	25–40 years	7380	20.8
	>40 years	1065	3
**Sex**	Male	23032	65
	Female	12403	35
**Occupation**	Student	25727	72.6
	Government Employed	4707	13.3
	Farmer	205	0.6
	Private Worker	2400	6.8
	Driver	176	0.5
	Defense Force	1919	5.4
	Unemployed	301	0.8
**Donor Type**	Volunteer	35248	99.5
	Replacement	187	0.5
**Blood group**	A	10390	29.3
	AB	2419	6.8
	B	8078	22.8
	O	14548	41.1
**Place of Donation**	Mobile sites	32667	92.2
	Center	2768	7.8
**No of Donation**	New	26115	73.7
	2–3 times	6647	18.8
	>3 (Regular type)	2673	7.5

### Trend of Sero-prevalence of transfusion transmissible infections

**Fig 1:**
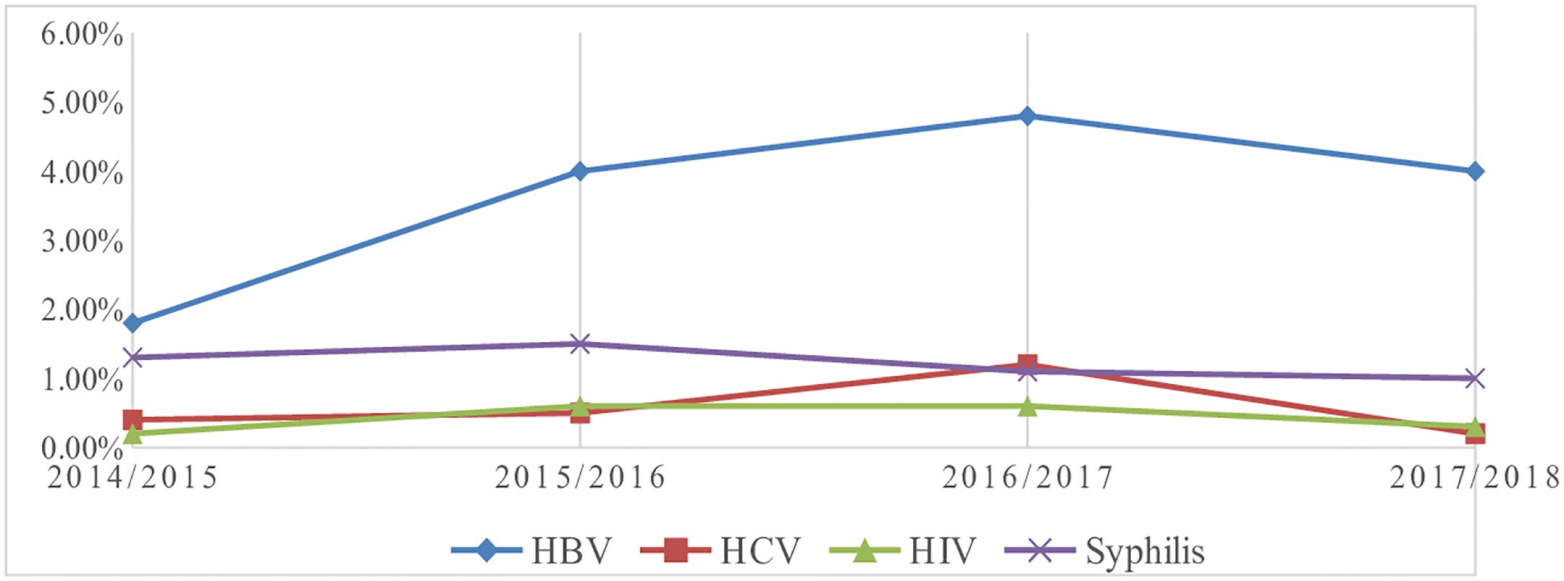
Graphical presentations of trends of TTI with respect to year of donation among blood donors at Bahir Dar district blood bank, Ethiopia 2007–2010.

From a total of 35,435 blood donors 2130 (6.0%) of them had serological evidence for at least one infection. The overall sero-prevalence of HBV, HCV, HIV and syphilis was 230 (6.0%) with 3.9%, 0.6%, 0.5% and 1.2% respectively. The total sero-prevalence of TTI showed an increment with respect to year with 183 in 2014/2015and 624 in 2017/2018. The sero-prevalence of HBV show a significant increment tend with respect to year of donation. On the other hand HCV and HIV sero-prevalence showed an increasing trend from 2014/2015to 2016/2017 and decrease in 2017/2018. The sero-prevalence of syphilis was 67 (1.3%) in 2014/2015 and duplicate in 2015/2016, 138 (1.5) but subsequently decrease to 110 (1.1%) in 2016/2017 intern it increases in 2017/2018which was 114 (1.0%) [(Table 2) and (Fig 1)].

**Table 2:** Prevalence of TTI with respect to donation year among blood donors at Bahir Dar district blood bank, northwest, Ethiopia.

Year of donation	No of Donors N (%)	Frequency of TTI N (%)				
		HBV	HCV	HIV	Syphilis	Total
**2014/2015**	5010 (14.1)	93 (1.8)	19 (0.4)	9 (0.2)	67 (1.3)	183 (3.6)
**2015/2016**	9421 (26.6)	377 (4.0)	45 (0.5)	56 (0.6)	138 (1.5)	601 (6.4)
**2018/2017**	9636 (27.2)	459 (4.8)	116 (1.2)	57 (0.6)	110 (1.1)	722 (7.5)
**2017/2018**	11368 (32.1)	460 (4.0)	23 (0.2)	38 (0.3)	114 (1.0)	624 (5.5)
**Total**	35435 (100)	1389 (3.9)	203 (0.6)	160 (0.5)	429 (1.2)	2130 (6.0)

TTI:—Transfusion Transmissible Infections, N:—Number/ Frequency

About 50 (0.14%) of donors were co-infected with more than one infection. From these majority of them were attributed to HBV-Syphilis 22 (44.0%) and 11 (22.0%) to HBV- HIV co-infection. one (2.0%) study participant was co-infected with HBV-HIV-Syphilis infection (Table 3).

**Table 3:** Prevalence of co-infections of HIV, HBV, HCV and syphilis among blood donors at Bahir Dar district blood bank, northwest, Ethiopia.

Co-infections	Number	Percent
**HBV- HCV**	6	12.0
**HBV-HIV**	11	22.0
**HBV- Syphilis**	22	44.0
**HCV- HIV**	5	10.0
**HCV- Syphilis**	1	2.0
**HIV- Syphilis**	4	8.0
**HBV- HCV- Syphilis**	1	2.0
**Total**	50	100

HBV:—Hepatitis B Virus, HCV:—Hepatitis C Virus, HIV:—Human Immunodeficiency Virus

### Prevalence and associated factors of HBV

The overall sero-prevalence of HBV was 1389 (3.9%). The prevalencewas4.3% in males and 3.2% in females. The age-specific distribution of HIV infection revealed that a high prevalence was detected among blood donors with in age group of > 40 years which shows 55 (5.2%) of sero-prevalence. In the bivariate binary logistic regression analysis, age, sex, occupation, Number and place of donation were significantly associated with HBV infection. However, in the multivariable analysis donors at the age of 25–40 years (AOR = 1.4; 1.2, 1.6), and > 40 years (AOR = 1.5; 1.1, 1.9), male blood donors (AOR = 1.3; 1.1, 1.4) and blood donors from zonal cities mobile site (AOR = 1.4; 1.1, 1.8) were at higher risk of HBV infection compared the reference groups. (Table 4).

**Table 4:** Logistic regression of HBV with sociodemographic characteristics of blood donors at Bahir Dar district blood bank, northwest, Ethiopia.

Variables		HBV Status (N)			COR (95% CI)	AOR (95% CI)
		Non-Reactive	Reactive	Total		
**Age**	18–24 Years	26016	974	26990	1.00	
	25–40 Years	7020	360	7380	1.4 (1.2, 1.5)	1.4 (1.2, 1.6)*
	>40 Years	1010	55	1065	1.5 (1.1, 1.9)	1.5 (1.1, 1.9)*
**Sex**	Female	12008	395	12403	1.00	
	Male	22038	994	23032	1.4 (1.2, 1.5)	1.3 (1.1, 1.4)*
**Occupation**	Student	24795	932	25727	1.00	
	Gov’t Employed	4462	245	4707	1.5 (1.3, 1.7)$	
	Farmer	193	12	205	1.6 (0.9, 3.0)	
	Own private work	2289	111	2400	1.3 (1.1, 1.6)$	
	Driver	170	6	176	0.9 (0.4, 2.1)	
	Defense force	1846	73	1919	1.1 (0.8, 1.3)$	
	Unemployed	291	10	301	0.9 (0.5, 1.7)	
**Place of donation**	Center	2675	93	2768	1.00	
	Regional city mobile site	17690	642	18332	1.0 (0.8, 1.3)	1.1 (0.8, 1.3)
	Zonal city mobile site	13681	654	14335	1.4 (1.1, 1.7)	1.4 (1.1, 1.8)*
**No of donation**	Repeated or Regular	9004	316	9320	1.00	
	New	25042	1073	26115	1.2 (1.1, 1.4)	1.1 (0.9, 1.3)*

^$^ indicates significance in bivariate but not in multivariate logistic regression analysis, and

*indicates significant variable with Pvalue less than 0.05 in multivariate logistic regression analysis.

### Prevalence and associated factors of HCV

The overall sero-prevalence of HCV among blood donors was 203 (0.6%). The distribution of the prevalence was 134 (0.6%) and 69 (0.6%) in males and in females respectively. In the bivariate binary logistic regression analysis, occupation, and place of donation were significantly associated with HCV infection. In the multivariable analysis donors government employed (AOR = 1.5; 1.0, 2.2), and unemployed (AOR = 3.8; 1.5, 9.4) by their occupation by their occupation were more likely to have HCV infection compared to student. On the other hand blood donors from regional (AOR = 2.3; 1.1, 4.9) and zonal cities mobile site (AOR = 2.5; 1.2, 5.5) were at higher risk of HCV infection compared those donate at the center. (Table 5).

**Table 5:** Logistic regression of HCV with sociodemographic characteristics of blood donors at Bahir Dar district blood bank, northwest, Ethiopia.

Variables		HCV Status (N)			COR (95% CI)	AOR (95% CI)
		Non-Reactive	Reactive	Total		
**Age**	18–24 Years	26845	145	26990	1.00	
	25–40 Years	7332	48	7380	1.2 (0.8, 1.7)	
	>40 Years	1055	10	1065	1.7 (0.9, 3.3)	
**Sex**	Female	12334	69	12403	1.00	
	Male	22898	134	23032	1.0 (0.8, 1.4)	
**Occupation**	Student	25590	137	25727	1.00	
	Gov’t Employed	4670	37	4707	1.5 (1.0, 2.1)	1.5 (1.0, 2.2)*
	Farmer	203	2	205	1.8 (0.4, 7.5)	3.3 (0.7, 14)
	Own private work	2389	11	2400	0.8 (0.5, 1.6)	0.9 (0.5, 1.8)
	Driver	176	0	176		
	Defense force	1908	11	1919	1.1 (0.6, 2.0)	1.0 (0.5, 1.9)
	Unemployed	296	5	301	3.1 (1.3, 7.7)	3.8 (1.5, 9.4)*
**Place of donation**	Center	2760	8	2768	1.00	
	Regional cities mobile site	18227	105	18332	1.9 (0.9, 4.1)	2.3 (1.1, 4.9)*
	Zonal cities mobile site	14245	90	14335	2.2 (1.1, 4.5)	2.5 (1.2, 5.5)*
**No of donation**	Repeated Or Regular (>3 times)	9270	50	9320	1.00	
	New	25962	153	26115	1.1 (0.8, 1.5)	

*indicates significant variable with Pvalue less than 0.05 in multivariate logistic regression analysis.

### Prevalence and associated factors of HIV

About 160 (0.5%) of blood donors have sero reactivity for HIV infection. The distribution of the prevalence was 106 (0.5%) and 54 (0.4%) in males and in females respectively. In the bivariate binary logistic regression analysis, occupation, and number of donation were significantly associated with HIV infection. While in multivariable analysis number of donation was significantly associated. Those blood donors who donate blood for the first time or new blood donors (AOR = 1.7; 1.1, 2.7) was at higher risk of HIV infection compared those regular type of blood donors. (Table 6).

**Table 6:** Logistic regression of HIV with sociodemographic characteristics of blood donors at Bahir Dar district blood bank, northwest, Ethiopia.

Variables		HIV Status (N)			COR (95% CI)	AOR (95% CI)
		Non-Reactive	Reactive	Total		
**Age**	18–24 Years	26875	115	26990	1.00	
	25–40 Years	7343	37	7380	1.2 (0.8, 1.7)	
	>40 Years	1057	8	1065	1.8 (0.9, 3.6)	
**Sex**	Female	12349	54	12403	1.00	
	Male	22926	106	23032	1.1 (0.8, 1.5)	
**Occupation**	Student	25625	102	25727	1.00	
	Gov’t Employed	4683	24	4707	1.3 (0.8, 2.0)	
	Farmer	203	2	205	2.5 (0.6, 10)	
	Own private work	2388	12	2400	1.3 (0.7, 2.3)	
	Driver	175	1	176	1.4 (0.2, 10)	
	Defense force	1901	18	1919	2.4 (1.4, 3.9)$	
	Unemployed	300	1	301	0.8 (0.1, 6.0)	
**Place of donation**	Center	2760	8	2768	1.00	
	Regional cities mobile site	18258	74	18332	1.4 (0.7, 2.9)	
	Zonal cities mobile site	14257	78	14335	1.9 (0.9, 3.9)	
**No of donation**	Repeated Or Regular (>3 times)	9293	27	9320	1.00	
	New	25982	133	26115	1.8 (1.2, 2.7)	1.7 (1.1, 2.7)*

^$^ indicates significance in bivariate but not in multivariate logistic regression analysis, and

*indicates significant variable with Pvalue less than 0.05 in multivariate logistic regression analysis.

### Prevalence and associated factors of syphilis

The overall sero-prevalence of syphilis infection among blood donors was 429 (1.2%). The prevalence was 1.5% in males and 0.7% in females. The age-specific distribution of HIV infection revealed that a high prevalence was detected among blood donors with in age group of > 40 years 107 (10.0%). Both in bivariate binary and multivariable logistic regression analysis age, sex and occupation were significantly associated with syphilis infection. Those blood donors at the age of 25–40 years (AOR = 1.5; 1.1, 2.1) and > 40 years (AOR = 8.2; 5.8, 11.7), male blood donors (AOR = 1.3; 1.0, 1.6), donors with occupation of government employed (AOR = 2.2; 1.6, 3.1), own private work (AOR = 2.5; 1.8, 3.7), defense force (AOR = 3.0; 2.1, 4.3)and unemployed (AOR = 2.9; 1.4, 6.1) were was at higher risk of syphilis HIV infection compared those reference group (Table 7).

**Table 7:** Logistic regression of syphilis with sociodemographic characteristics of blood donors at Bahir Dar district blood bank, northwest, Ethiopia.

Variables		Syphilis Status (N)			COR (95% CI)	AOR (95% CI)
		Non-Reactive	Reactive	Total		
**Age**	18–24 Years	26813	177	26990	1.00	
	25–40 Years	7235	145	7380	3.1 (2.4, 3.8)	1.5 (1.1, 2.1)*
	>40 Years	958	107	1065	17 (13.2, 21.7)	8.2 (5.8, 11.7)*
**Sex**	Female	12311	92	12403	1.00	
	Male	22695	337	23032	2.0 (1.6, 2.5)	1.3 (1.0, 1.6)*
**Occupation**	Student	25574	153	25727	1.00	
	Gov’t Employed	4576	131	4707	4.8 (3.8, 6.1)	2.2 (1.6, 3.1)*
	Farmer	201	4	205	3.3 (1.2, 9.1)	0.9 (0.3, 2.5)
	Own private work	2328	72	2400	5.2 (3.9, 6.7)	2.5 (1.8, 3.7)*
	Driver	175	1	176	0.9 (0.1, 6.9)	0.5 (0.1, 3.4)
	Defense force	1859	60	1919	5.4 (4.0, 7.3)	3.0 (2.1, 4.3)*
	Unemployed	293	8	301	4.6 (2.2, 9.4)	2.9 (1.4, 6.1)*
**Place of donation**	Center	2740	28	2768	1.00	
	Regional cities mobile site	18133	199	18332	1.1 (0.7, 1.6)	
	Zonal cities mobile site	14133	202	14335	1.4 (0.9, 2.1)	
**No of donation**	Repeated Or Regular (>3 times)	9212 25794	108	9320	1.00	
	New	25794	321108	26115	1.1 (0.8, 1.3)	

*indicates significant variable with Pvalue less than 0.05 in multivariate logistic regression analysis.

## Discussion

The current study tried to figure out the seroprevalence and trend of TTI among blood donors at Bahir Dar district blood bank, Ethiopia from 2007 to 2010 E.C or from July 2014 (2014/2015–June 2018 (2017/2018). The result showed that the overall sero-prevalence of TTI was 6.0%. The finding in line with previous reports from Pakistan 5.29% [10] and reports from Gondar 6.55% [6], Yirgalem, Hawassa7.1% [11] and Harar 6.6% [12] regions of Ethiopia. It was also relatively a low prevalence rate compared to previous reports from different African countries such as Tanzania 15.9% [13], Burkina Faso 29.82% [4], Ghana 18.9% [14], Nigeria ranging from 28.8% to 14.96% [15, 16], and Ethiopian studies from Gondar 9.5% [5] and Jijiga 11.5% [7]. On the other hand the current result was higher than study in India with sero-prevalence ranging from 0.58% to 2.73 [17–19], and Ethiopian studies from North Showa, central Ethiopia 2.4% [20], South west, Ethiopia3.61% [21]. In general variation in the total sample size, donor recruitment (proportion of volunteer to replacement donors), time period, strength of preliminary screening of donors and factors related to test algorithms used for screening, the test kits on the market, storage and validation of the test kits might be the possible reason for the discrepancy in the total seroprevalence of TTI between various studies.

In the current study the total sero-prevalence of TTI showed an increment with respect to year 3.6% in 2014/2015 to 5.5% in 2017/2018 even though it doesn’t show a trend. Consistently an increment in the sero-prevalence had been reported previous from Gondar study which was conducted from 2010–2012 [6]. This might be as a result of overall increment in the sero-prevalence of these TTI in the community since it is assumed that blood donors are a representative of the community. In addition it might be as a result of the implementation of sensitive diagnostic test methods for screening of TTI.

Hepatitis B virus infection has shown an intermediate or high endemicity level in low-income countries over the last five decades. Africa is on the whole considered to have a high HBV endemicity. HBV infection is hyper-endemic with prevalence of > 8% of hepatitis B surface antigen chronic carriers in the general population [22]. In the current study the prevalence of HBV infection among blood donors was 3.9%. Similar finding had been reported from Dire Dawa 3.73% [23]. On the other hand as compared to reports from other African countries; Tanzania 8.8% [13], Burkina Faso 14.96% [4], and Ghana 9.6% [14] the sero-prevalence in the current study was very low. More over the prevalence from the current study was higher than report from Egypt 2.3% [24]. The possible reason for these discrepancy in the sero-prevalence across studies might be due to difference in socio-demographic, socio-economic status, cultural and societal behavior, risk factors, and awareness of the population. In additions, strength of preliminary screening of donors and methods of laboratory diagnosis used for screening can also be the possible reasons.

Our study revealed that males (AOR = 1.3; 95% CI: 1.1, 1.4) were more likely to have HBV infection than females. A consistent result have been reported from number of studies from different regions of Ethiopia [5, 6, 23, 25] and studies from other country such as Ghana [14], Egypt [24]. This might be due to the fact sex is the genetic determinant of the disease outcome. When HBV infection is acquired during adult life, 95% of subjects will clear the infection and develop protective antibodies (HBsAb). However, in 5% to 10% of subjects, the virus will establish a persistent infection. With this regard males are approximately 1.5 times more likely to develop chronic HBV infection than females as a result of the slower plasma disappearance rate for HBsAg in males compared to females [26]. More over gender difference in behavioral risk factors such as having multiple sex partner could attribute to an increased in the prevalence of HBV among male donors.

According to the current study the overall sero-prevalence of HCV among blood donors was 0.6%. The result in line with previous studies from different regions of Ethiopia, Gondar ranging from 0.7%–0.8% [5, 6], Bahir Dar 0.63% [27], Hawassa 0.6% [11], Harar 0.8 [12] and other country 0.86% in China [28]. On the other hand this finding was higher as compared to previous studies in southwest, Ethiopia 0.2% [29] and other countries such as 0.09%–0.34% in India [17, 19], and 0.06% in Pakistan [30]. In contrast this finding was lower as compared to study from Burkina Faso 8.7% [4], Ghana 4.4% [14], and Nigeria 3.6% [31]. The possible reason for variation in the prevalence of HCV infections across studies might be due to differences in risk behaviors such as injecting drug use and sharing objects for skin piercing between different geographical areas, strength of preliminary screening of donors and screening method.

According to the current study unemployed blood donors (AOR = 3.8; 1.5, 9.4) were more likely to have HCV infection compared to student. This might be due to low socio-economic levels of unemployed donors, as they are most likely to indulge in risky sexual relationships that may expose them to TTIs. Moreover, as a consequence of economic problems, unemployed donors may experience risky practices, such as sharing of personal care items toothbrushes, sharing of sharp kitchen materials, and having sexual contact with a person infected with TTIs. Employed donors were also at higher risk of HIV-infection compared to student donors. Even though the reason is not clear it might be due to engagements in more risky behaviors over time and the transfusion of unsafe blood and/ or blood products in their lifetime.

The current study revealed that the overall sero-prevalence of HIV among blood donors 0.5%. the finding was consistent with previous studies from other part of Ethiopia specifically Jijiga 0.4% [12], Gondar 0.7% & 0.8% [5, 6] Harar 0.8% [12] and other countries such as India 0.34% [19] china 0.31% [28]and Ghana 4.9% [14]. On the other hand the sero-prevalence was low than studies from other countries such as Nigeria 3.1% [31], and Tanzania 3.8% [13].

The current study also showed that those blood donors who donate blood for the first time or new blood donors (AOR = 1.7; 1.1, 2.7) were more likely to have HIV infection compared those regular type of blood donors. Similar finding have been reported from Kenya study [32]. This might be directly associated with post donation counseling service a failure to inform blood donors regarding their HIV positive status does more harm than good. There are individual who had a wrong perceptions of donating blood as a method of knowing once own status regarding TTI and continue their life as normal if they are not informed so that this will in turn increases the transmission of HIV among the general populations by one and other means.

The prevalence syphilis in our study was 429 (1.2%). A consistent result have been reported from to Kenya 1.0% [32], while it was lower than previous studies from Tanzania 4.7% [13], Burkina Faso 3.96% [4], Nigeria 4.2% [31]. In contrast the current finding a higher prevalence was reported from previous studies in Jijiga 0.1% [7], south west regions of Ethiopia 0.14% [29], India ranging 0.06–0.3% [19, 33], and Pakistan study 0.43% [30].

According to the current study male blood donors (AOR = 1.3; 1.0, 1.6) donors with occupation defense force (AOR = 3.0; 2.1, 4.3) and unemployed (AOR = 2.9; 1.4, 6.1) were more likely to have syphilis infection. Similar finding have been reported study from North Showa [34] and Gondar [5]. This could be attributed to the prevalent risk behaviors such as having multiple sex partners and alcohol abuse among males than females. The relatively higher syphilis sero-positivity among defense force might be attributed to poor awareness regarding mode of transmission due to their low educational status in which these individuals might have sexual contact with a person infected with these type of TTI. More over low socio-economic levels of unemployed donors can be the attributing factor for the high prevalence of the disease among these group since as a consequence of economic problems, unemployed donors may experience risky practices, such as sharing of personal care items toothbrushes, sharing of sharp kitchen materials, and having sexual contact with a person infected with TTIs. On the other hand those blood donors at the age of 25–40 years (AOR = 1.5; 1.1, 2.1) and > 40 years (AOR = 8.2; 5.8, 11.7) were more likely to have syphilis infections compared to those at the age of 18–24 years. This might be due to the fact that most of blood donors in our study were in the ages of 18–24 years and university students whom they are considered as have good aware of regarding these infections.

More over the current study tried to figure out the prevalence of co-infections of TTI among blood donors. Our result revealed that 50 (0.14%) of the study participants co-infected with more than one infection. Previous studies from different regions of Ethiopia showed the prevalence of co-infection of TTI 0.062%–0.8% in Gondar [5, 6] and African countries such as 3.3% in Burkina Faso [4], 3.42% in Ghana [14], and 1.8% in Tanzania [13]. According our result from those who had been confirmed as having co-infection majority of them were attributed to HBV-Syphilis 22 (44.0%) and 11 (22.0%) to HBV- HIV co-infection. Several studies had also reported co-infections of HBV-HIV, HBV- HCV, HBV- Syphilis and HIV- Syphilis as commonly encountered one [4–6, 12–14]. The possible reason for the occurrence of co-infections could be because these infections share similar modes of transmission. This high rate of co-infection might be due to the fact that these pathogens share common modes of transmission and risk groups.

Retrospective nature of the study might be considered as it limitation which render from inclusions of all risk factors associated with TTIs. Though we tried to demonstrate the trend and sero-prevalence of the major TTIs among blood donors at Bahir Dar district blood bank.

## Conclusion and recommendations

Despite stringent donor screening and testing practices, safe blood free from TTIs remains an elusive goal since the prevalence of TTI is substantial increased overtime. This finding showed growing evidence in the burden of TTIs in blood donors which is directly related to the prevalence in the general population thus it requires community-based studies to identify societal risk factors. Age, sex, occupation and number of donation significantly associated with different type of TTIs. Thus advanced and vigilance screening of donated blood should be there prior to transfusion. In additions, there should be strategies for monitoring the implementation of post donation counseling for recruitment and retention of safe regular donors is the need of the time. Further prospective studies should be conducted rigorously with advanced methods and subclinical infections need to be conducted in order to identify the cause-effect relationship of TTIs with its contributing factors.
